# Fine tuning of the temporal expression of HTLV-1 and HTLV-2

**DOI:** 10.3389/fmicb.2013.00235

**Published:** 2013-09-02

**Authors:** Ilaria Cavallari, Francesca Rende, Cecilia Bender, Maria G. Romanelli, Donna M. D'Agostino, Vincenzo Ciminale

**Affiliations:** ^1^Department of Surgery, Oncology and Gastroenterology, University of PadovaPadova, Italy; ^2^Department of Life and Reproduction Science, University of VeronaVerona, Italy; ^3^Istituto Oncologico Veneto, Istituto di Ricovero e Cura a Carattere ScientificoVeneto, Italy

**Keywords:** HTLV-1, HTLV-2, splicing, Tax, Rex

## Abstract

Human T-cell leukemia virus types 1 and 2 (HTLV-1 and HTLV-2) are delta retroviruses that share a common overall genetic organization, splicing pattern, and ability to infect and immortalize T-cells *in vitro*. However, HTLV-1 and HTLV-2 exhibit a clearly distinct pathogenic potential in infected patients. To find clues to the possible viral determinants of the biology of these viruses, recent studies investigated the timing of expression and the intracellular compartmentalization of viral transcripts in *ex-vivo* samples from infected patients. Results of these studies revealed a common overall pattern of expression of HTLV-1 and -2 with a two-phase kinetics of expression and a nuclear accumulation of minus-strand transcripts. Studies in cells transfected with HTLV-1 molecular clones demonstrated the strict Rex-dependency of this “two-phase” kinetics. These studies also highlighted interesting differences in the relative abundance of transcripts encoding the Tax and Rex regulatory proteins, and that of the accessory proteins controlling Rex expression and function, thus suggesting a potential basis for the different pathobiology of the two viruses.

## Introduction

Human T-cell leukemia virus types 1 and 2 (HTLV-1 and HTLV-2) are genetically related deltaretroviruses (Lairmore and Franchini, 2007). Although both viruses immortalize T-cells in culture and establish a persistent infection *in vivo* (Matsuoka and Jeang, 2007), they differ substantially in terms of pathogenic potential. Unlike HTLV-1, which causes adult T-cell leukemia/lymphoma (ATLL) and tropical spastic paraparesis/HTLV-1-associated myelopathy (TSP/HAM), HTLV-2 has not been linked to lymphoid malignancies. However, HTLV-2 infection is associated with an increase in lymphocytes counts (Bartman et al., 2008) and coinfection with HTLV-2 plays an important role in the progression of HIV-infected patients to AIDS (Casoli et al., 2007).

The two viruses present similar genetic organization and expression strategies (Ciminale et al., 1992, 1995; Koralnik et al., 1992; Cavallari et al., 2011) and share an average 65% identity at the nucleotide level, with higher conservation in the gag, pol, env, and tax/rex genes and lower in the long terminal repeats (LTR), protease and proximal pX region.

The coding potential of the HTLV genomes is greatly enhanced by several gene expression strategies that include ribosomal frameshifting (which generates a Gag-Pro-Pol polyprotein) and alternative splicing (which produces distinct mRNAs coding for Env and non-structural proteins coded by the X region). The pX regions of HTLV-1 and HTLV-2 contain, respectively, four and five major open reading frames (ORFs), termed x-I through x-V. The x-III and x-IV ORFs code for Rex and Tax, which are produced from a dicistronic doubly-spliced mRNA containing exons 1, 2, and 3. Tax and Rex play a critical role in regulating viral expression at the transcriptional and post-translational levels, respectively. Other transcripts produced by the X region code for accessory proteins; these transcripts include singly-spliced mRNAs coding for p21rex, p12, and p13 (HTLV-1), truncated isoforms of Rex (tRex) and p28 (HTLV-2) and doubly-spliced mRNAs coding for p30/tof (HTLV-1), p10 and p11 (HTLV-2) (Ciminale et al., 1992, 1995; Koralnik et al., 1992). While most of the regulatory and accessory proteins of the two viruses share structural and functional homologies, p13 and p8 appear to be unique to HTLV-1 and p11 is unique to HTLV-2. p13 corresponds to the C-terminal 87 amino acids of p30/tof (Koralnik et al., 1992) and is localized mainly in the mitochondrial inner membrane (Ciminale et al., 1999) although it can be partly localized to the nucleus especially when expressed at high levels and in conjunction with Tax (Andresen et al., 2011). p13 increases mitochondrial permeability to K^+^ and activates the electron transport chain, resulting in an increased production of mitochondrial reactive oxygen species (ROS) (Silic-Benussi et al., 2009). Increased ROS affect both cell survival and proliferation depending on the cell's inherent ROS set-point. Results of our studies indicated that p13 expression leads to in mitogenic activation of normal resting T-cells, which have low ROS levels; in contrast, p13 induces cell death in cancer cells which are characterized by a high ROS setpoint (Silic-Benussi et al., 2010a, b). p8, which is a cleaved form of p12, traffics to the immunological synapse and favors T-cell anergy. p8 also increases cell-to-cell viral transmission through the formation of intercellular conduits among T-cells (Van Prooyen et al., 2010a, b). p11, which is unique to HTLV-2, exhibits a partial functional homology to HTLV-1 p12, in that it is able to bind the MHC heavy chain (Johnson et al., 2000).

Recent studies showed that both HTLV-1 and HTLV-2 also produce complementary-strand mRNAs that are transcribed from promoters in the 3′ LTR; these genes were termed, respectively, HBZ (HTLV-1 bZIP factor) (Gaudray et al., 2002) and APH2 (anti-sense protein of HTLV-2) (Halin et al., 2009). HTLV-1 generates two major minus-strand transcripts, one spliced (hbz sp1) and the other unspliced (hbz us) (Cavanagh et al., 2006; Murata et al., 2006; Satou et al., 2006), translated in two proteins that differ by 7 amino acids at their N-terminus (Murata et al., 2006). The negative strand of HTLV-2 generates only one spliced transcript that codes for the APH2 protein (Halin et al., 2009). Both HBZ and APH2 interact with CREB, resulting in the inhibition of Tax-mediated transcription from the 5′LTR (Gaudray et al., 2002; Halin et al., 2009); however, while the hbz mRNA has a growth-promoting effect on T-cells (Satou et al., 2006), no function has been described for the aph2 mRNA. Interestingly, the hbz and aph2 transcripts were detected mainly in the nucleus of infected cells (Rende et al., 2011; Bender et al., 2012), supporting the notion of their possible role as non-coding RNAs.

## Temporal analysis of HTLV-1 expression

An early study by Hidaka et al. (Hidaka et al., 1988) established that HTLV-1 expression is controlled by two key regulatory circuits: a positive feedback provided by the viral transactivator Tax, which drives transcription of the viral genome, and a post-transcriptional regulatory loop provided by Rex, which binds to the Rex-responsive element (RXRE) present at the 3′ end of HTLV-1 transcripts and enhances the nuclear export and expression of a subset of mRNAs coding for the virion-associated proteins Gag-Pol and Env. This study was conducted by transfecting wild type and Rex-mutant proviral clones in fibroblast cell lines followed by isolation of nuclear and cytoplasmic mRNAs and analysis by Northern Blot, a technique that was not capable of distinguishing among different mRNAs of similar size. This approach revealed three major size classes of polyadenylated mRNAs: (i) the 9-kb genomic mRNA coding for Gag-Pro-Pol, (ii) the 4-kb singly spliced mRNA coding for the envelope glycoproteins, (iii) mRNAs of approximately 2.1 kb coding for Tax and Rex (Hidaka et al., 1988). The study did not consider other spliced mRNAs coding for the accessory proteins, as those were discovered several years later. The authors also observed a distinct timing of expression of the transcripts, with the 2.1 kb mRNA preceding the expression of the 9- and 4-kb transcripts, while at a later stage only a trace of the 2.1 kb mRNA, was detectable. Interestingly, a Rex-defective proviral clone only expressed the 2.1 kb mRNAs, demonstrating that Rex is essential for gag and env (but not tax/rex) expression.

A subsequent study by Kimata and Ratner (1991) investigated the temporal regulation of HTLV-1 expression following infection of primary lymphocytes by co-cultivation with the lethally-irradiated HTLV-1-infected cell line MT2. Viral transcripts were detected by RT-PCR using primers spanning the splice-junctions of known viral mRNAs (gag, env, tax/rex). Results showed that tax/rex mRNA appeared at day 4 of co-cultivation, the env mRNA at day 25, and the gag–pol mRNA at day 100 of co-cultivation. The expression of viral transcripts were monitored for 150 days post-infection.

With the aim of investigating the timing of expression of all the transcripts encoded by HTLV-1, Li et al. employed RT-PCR and both an *in vitro* model and a rabbit animal model of HTLV-1 infection (Li et al., 2009). Results showed a general trend toward increased viral gene expression over time both *in vitro* and *in vivo*. However, these studies did not reveal a clear temporal separation between early and late viral transcripts or a switch in the expression of early vs. late viral transcripts as suggested by Hidaka et al. (1988).

To gain further insight into these aspects of HTLV-1 regulation, we recently investigated the timing of spontaneous onset of HTLV-1 expression *in vitro* in primary cells obtained from infected individuals, and measured the levels of individual transcripts by real-time RT-PCR (qRT-PCR) using splice site-specific primers, which allowed measurements of the levels of individual alternatively spliced transcripts. qRT-PCR analysis was also carried out in cells transfected with wild type and Rex-knockout HTLV-1-molecular clones.

We first employed an *ex vivo* viral reactivation model based on the depletion of CD8+ cytotoxic T cells from peripheral blood mononuclear cells (PBMC) of HTLV-1-infected patients, which results in a sharp rise in viral expression (Hanon et al., 2000). Results obtained for 6 TSP/HAM and 3 ATLL patients revealed that the most abundant plus-strand transcripts were tax/rex, gag, and env, followed by p21rex, p30tof, p13, and p12 mRNAs; minus-strand HBZ transcripts were expressed at high levels (Rende et al., 2011).

**Figure 1:**
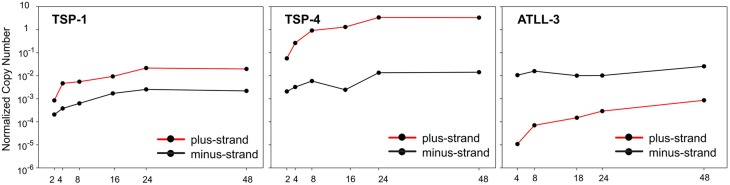
**Normalized Copy Number (NCN) of the sum of all plus-strand viral mRNAs (red lines) and of the minus-strand mRNAs, (hbz sp1 and us) (black lines) in PBMCs from 3 infected patients (TSP-1, TSP-4, ATLL-3) over a 48-h time course of culture *in vitro***.

These studies also revealed a sharp upregulation of viral expression upon *ex vivo* culture which is shown in Figure 1, where the time course of expression of all plus-strand viral mRNAs (NCN, Normalized Copy Number) is compared to the NCN of all negative-strand mRNAs (hbz sp1 and us). Results of this analysis in samples examined from the 9 patients revealed 3 main patterns (Figure 1). The minus-strand transcripts were slightly up-regulated and their overall NCN values were very similar among the different patients. In contrast, the up-regulation of plus-strand transcripts was more evident and the expression levels of plus-strand transcripts were highly variable in these patients. Patient TSP-4 shows an overall expression of plus-strand NCN of about 2 orders of magnitude greater than TSP1, instead ATLL-3 patient showed a peculiar pattern of expression, with a marked prevalence of minus-strand transcripts compared to plus-strand transcripts (only tax/rex, gag, and env mRNAs were detectable). This patient, whose PBMCs were dominated by monoclonal leukemic cells, might thus represent an example of the subset of ATLL cases in which plus-strand transcription is inhibited by genetic or epigenetic mechanisms (Taniguchi et al., 2005).

**Figure 2:**
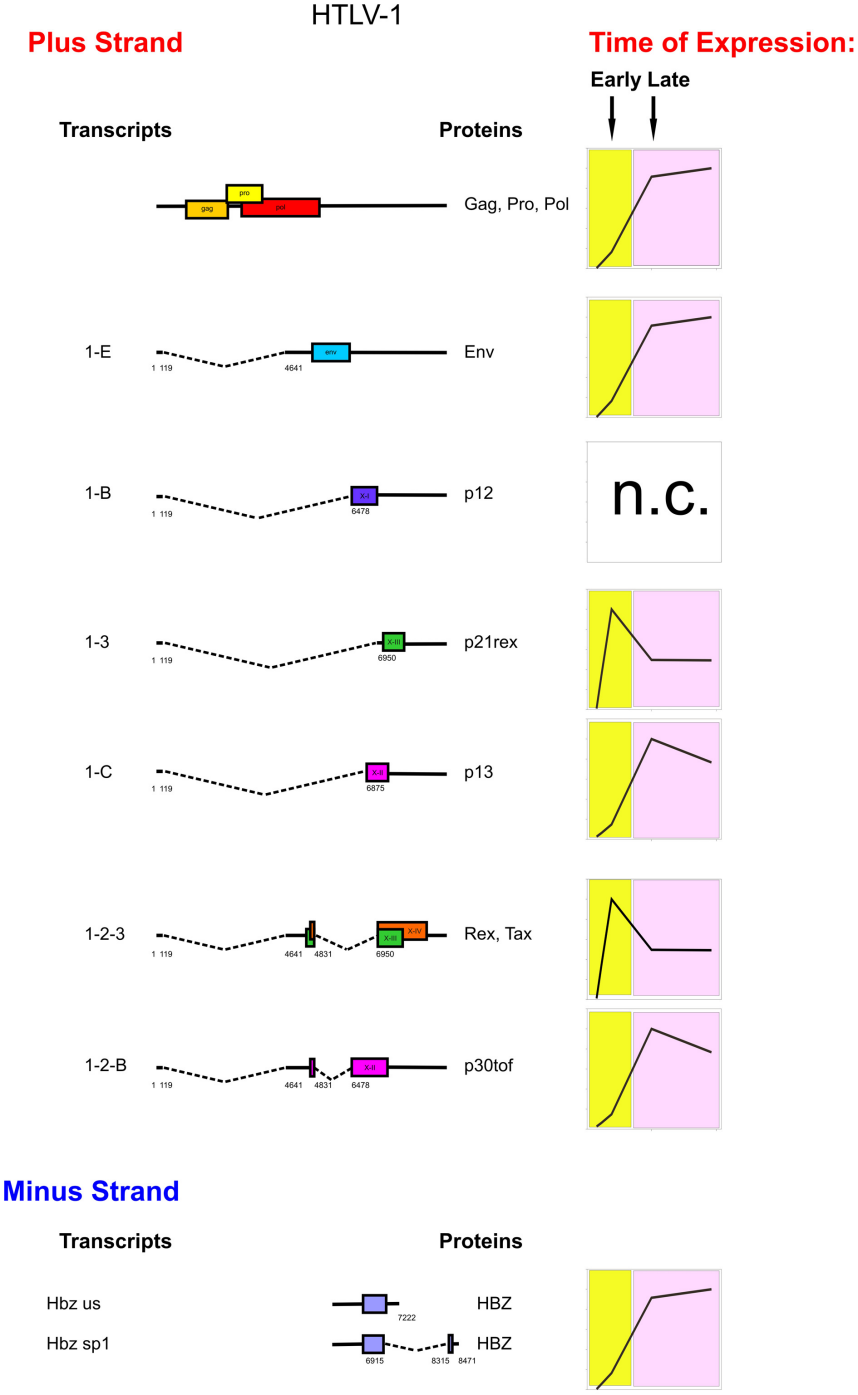
**Genetic organization, splicing pattern and kinetics of expression of HTLV-1 mRNAs.** On the left, structure and coding potential of HTLV-1 alternatively spliced mRNAs are shown. ORFs are indicated by boxes. Splice sites are indicated by numbers. Right-hand graphs show schematic representations of the prevalent kinetics of expression of HTLV-1 mRNAs in different patients. The p12 mRNA did not show a consistent pattern of expression among the different patients (n.c.).

The graphs in Figure 2 illustrate the prevailing pattern of the kinetics of expression of individual HTLV-1 mRNAs revealed by these studies. Tax/rex was the earliest transcript in all patients and p21rex also showed an early peak of expression in the majority of the patients; env, gag, p13, p30tof, and hbz mRNAs revealed a late peak of expression in the majority of the cases. Instead, p12 showed a very variable pattern of expression in the patients. This distinct temporal expression pattern is consistent with the tax/rex mRNA acting as an “early” master gene that drives the expression of the other viral transcripts (Rende et al., 2011). Nevertheless, this early-late switch in viral expression is apparently absent in the ATLL-3 patient, while it was evident in all the other patients examined.

This study also tested the HTLV-1 mRNA expression using an *in vitro* model based on the transfection of full-length HTLV-1 molecular clones, which made it possible to investigate the nucleo-cytoplasmic partition of the different transcripts during the time course of expression and to assess the effects of Rex on these processes. Consistent with the results obtained from patient PBMCs, transfection of wild-type HTLV-1 resulted in an “early” nucleo-cytoplasmic export of tax/rex followed by a rise in the export of gag and env mRNAs. Interestingly, this two-phase kinetics was not observed upon transfection of a REX-KO HTLV-1 molecular clone, demonstrating the strict Rex-dependency of this “two-phase” kinetics of expression (Rende et al., 2011). The early expression timing of tax/rex suggests that expression of this transcript is independent of Rex. However, two studies based on the expression of intronless cDNAs derived from the full-length HTLV-1 mature transcripts indicated that Rex may enhance the expression of the tax/rex RNA (D'Agostino et al., 1999; Bai et al., 2012) and binds the RXRE in the context of this transcript (Bai et al., 2012). These apparently conflicting results suggest that the impact of Rex is highly dependent on the processing pathway of the transcripts. It is thus possible that routing the transcripts through the splicing machinery might result in increased efficiency of expression even in the absence of Rex.

## Temporal analysis of HTLV-2 expression

Like HTLV-1, HTLV-2 produces plus- and minus-strand alternatively spliced transcripts that code for virion components and non-structural proteins, including Tax, Rex, and the accessory proteins (Feuer and Green, 2005; Younis and Green, 2005).

The kinetics of expression of the individual HTLV-2 transcripts were described for the first time by Bender et al. (2012). In this paper, the authors investigated the expression kinetics of HTLV-2 mRNAs in a chronically infected cell line and in PBMCs obtained from 3 infected patients using splice site-specific primers and qRT-PCR. The three patients analyzed had similar proviral loads and were HTLV-1- and HIV-1-negative (Bender et al., 2012).

**Figure 3:**
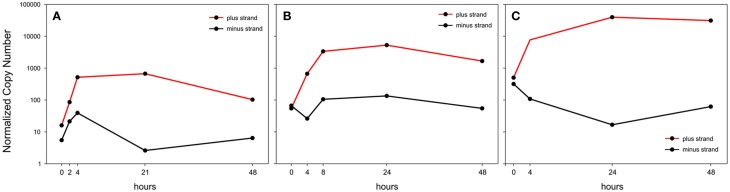
**Normalized Copy Number (NCN) of the sum of all plus-strand viral mRNAs (red line) and of the minus-strand mRNA, aph2 (black line) in PBMCs from 3 HTLV-2-infected patients (A,B and C) in a 48-h time course of culture *in vitro***.

Upon *ex vivo* culture of PBMC the expression of plus-strand transcripts was sharply upregulated in all the examined patients, although the overall expression levels at time zero were highly variable, with patient A showing much lower levels of expression compared to patients B and C (Figure 3). On the other hand, the minus-strand transcript (APH2) was transiently upregulated in patient A, not significantly changed in patient B and strongly downregulated (>20-fold) in patient C, who exhibited the highest levels of plus-strand mRNAs expression. The patients thus showed an apparent inverse correlation between changes in minus-strand expression and levels of expression of plus-strand transcripts (Figure 3).

**Figure 4:**
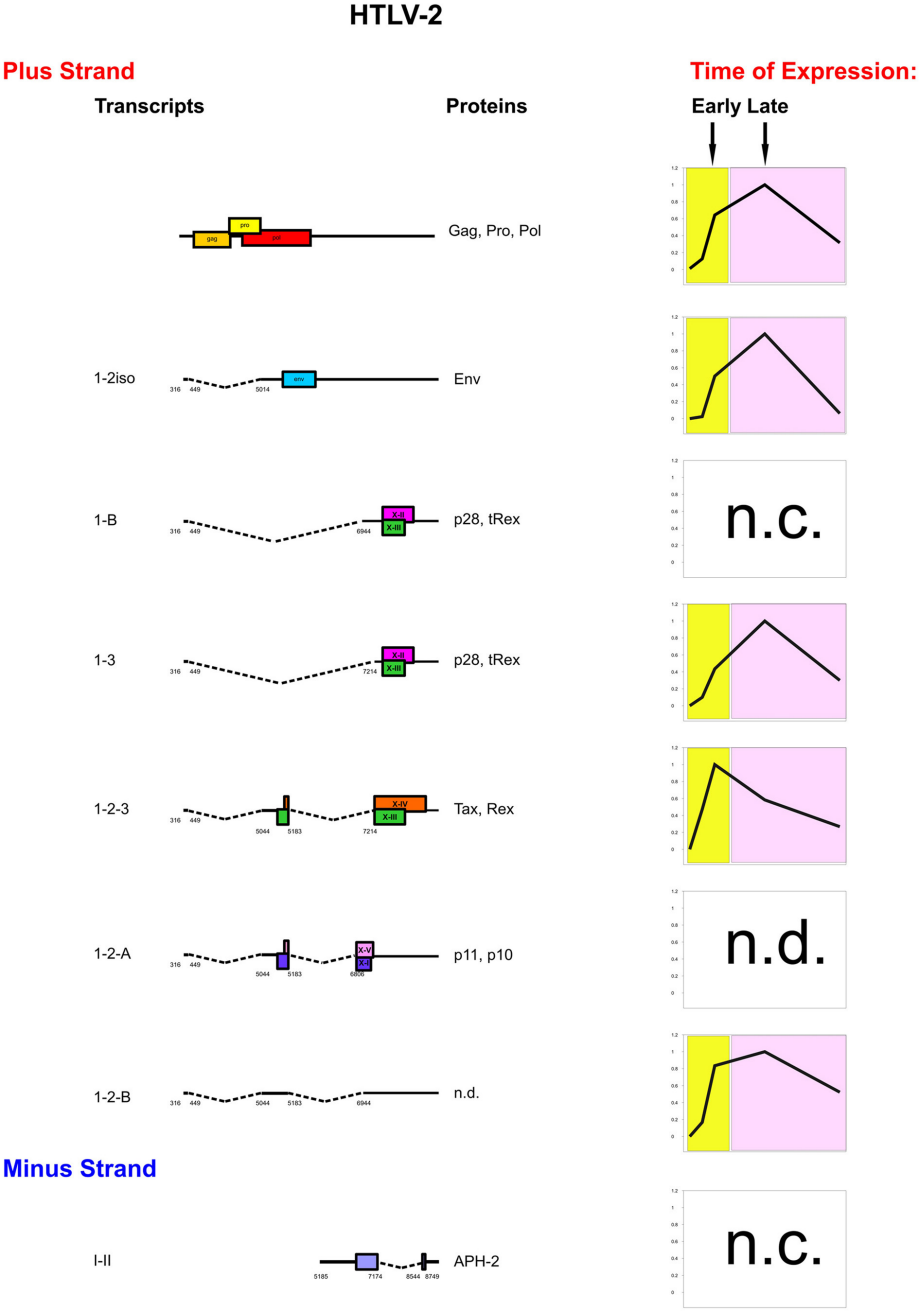
**Genetic organization, splicing pattern and kinetics of expression of HTLV-2 mRNAs.** The structure and coding potential of HTLV-2 alternatively spliced mRNAs are shown on the left. ORFs are indicated by boxes. Splice sites are indicated by numbers. The graphs depict the prevalent kinetics of expression of HTLV-2 mRNAs in different patients. n.c. indicates transcripts that did not show a consistent pattern of expression in different patients. n.d.: not determined.

Figure 4 shows a synoptic representation of the kinetics of expression of HTLV-2 mRNAs. Patients A and B showed a two-phase kinetics of HTLV-2 gene expression, with an early sharp rise in tax/rex(1-2-3) expression followed by a later expression of gag, env(1-2iso), p28/tRex(1-3), and 1-2-B. One of the two patients also expressed p28/tRex(1-B) as an early transcript, although at a level 1000-fold lower than the “late” 1-3 transcript that also encodes p28 and tRex (Bender et al., 2012). Patient C showed a peculiar kinetics of expression with the APH2 expressed only at the beginning of the time-course followed by a sharp downregulation, while tax/rex(1-2-3), gag, env(1-2iso), and p28/tRex(1-3) were expressed as later transcripts. The p11/p10(1-2-A) mRNA was below the threshold of detection in all patients, suggesting that these proteins might play a marginal role, at least in the context of the infected cells circulating in the peripheral blood.

To verify these findings, we employed an *in vitro* model in which viral expression in the BJAB-Gu HTLV infected cell line was synchronized by controlling the conditions of *in vitro* culture. Diluting a culture of confluent cells, resulted in an overall upregulation of viral gene expression. Results of qRT-PCR confirmed the 2-phase kinetics with the expression of the tax/rex(1-2-3), p28/tRex(1-B), and 1-2-B preceding that of the gag/pol, env, and p28/tRex(1-3) mRNAs.

## Molecular basis for the 2-phase kinetics and regulation of rex function by viral proteins

To find clues to the possible regulatory mechanism underlying the 2-phase kinetics of HTLV-1 expression, we used the expression data to generate a mathematical model (Corradin et al., 2011; Rende et al., 2011). Results underscored the importance of a delay in Rex function compared with Tax in the observed expression kinetics. These considerations led us to investigate the time course of Tax and Rex protein expression from the ACH full-length infectious molecular clone and from a plasmid coding for the mature *tax/rex* mRNA. Flow cytometry analyses showed a relative accumulation of Rex at later time points; consistent with this finding, Rex revealed a slower rate of degradation compared with Tax, suggesting that the activity of these two regulatory proteins might be controlled at the post-translational level. Similar experiments have not yet been carried out for HTLV-2.

A further layer of complexity of the HTLV regulatory networks is revealed by experimental evidence indicating that the function of Rex might be controlled by virus-encoded inhibitors. Among these, truncated forms of Rex lacking the N-terminal, RXRE-binding domain are produced by singly spliced transcripts both in HTLV-1 and HTLV-2 (Ciminale et al., 1996; Heger et al., 1999). These truncated Rex isoform are termed p21Rex (HTLV-1) and tRex (HTLV-2). tRex proteins contain the activation and multimerization domains of full-length Rex and act as inhibitors of full-length Rex-2 (Ciminale et al., 1997), while p21 Rex contains the activation but not the multimerization domains of Rex and its function as a repressor of the full length protein is controversial; some reports suggest that it inhibits Rex (Heger et al., 1999) whereas others do not (Ciminale et al., 1997; Bai et al., 2012).

The x-II ORF products of HTLV-1 (p30/tof) and HTLV-2 (p28) were shown to inhibit both the tax/rex mRNA and the Rex protein (Nicot et al., 2005) (see also the paper by Anupam at al. in this issue).

Interestingly, the relative expression of the tax/rex mRNA compared to the mRNAs coding for potential inhibitors of Tax (p30tof and p28) and Rex (p21rex and tRex) appears to be skewed toward tax/rex in HTLV-1 and toward the inhibitors in HTLV-2 (Rende et al., 2011; Bender et al., 2012). Furthermore, the main trex mRNA was expressed as a “late” transcript in HTLV-2 infected individuals while the mRNA coding for its HTLV-1 ortholog (p21rex) was expressed early in most HTLV-1 infected patients. These findings support the idea that complex feedback regulatory loops control HTLV expression at the post-transcriptional level and suggesting interesting differences in the fine-tuning of Rex function between the two viruses.

Future studies should be aimed at further investigating the functions of these regulatory proteins in HTLV-1 and HTLV-2 and at understanding how they may contribute to the different pathobiology of the two viruses.

## Conclusions and perspectives

The temporal regulation of viral expression has been thoroughly characterized in DNA tumor viruses such as herpes viruses, where different patterns of viral gene expression are associated with latent or productive phases of the viral life cycle and with different diseases [reviewed in Young and Rickinson (2004)]. In the case of EBV, the switch between early and late viral genes is achieved mainly through the genetic and epigenetic regulation of alternative viral promoters. Human papillomaviruses (HPV) exploit a combination of alternative promoter usage, alternative splicing and selection of “early” vs. “late” polyadenylation sites [reviewed in Schwartz (2008)]. The early/late pattern of HPV expression is associated with tissue tropism and restriction of the lytic cycle to differentiated epithelia (Moody and Laimins, 2010).

HTLV-1 and HTLV-2 encode structural, regulatory, and accessory genes from several alternatively spliced mRNAs generated both from the 5′ LTR and 3′ LTR promoters. This complex expression pattern is regulated by two key feedback loops mediated by the two regulatory proteins Tax and Rex which produce a two-phase kinetics in both viruses.

The fact that both HTLV-1 and HTLV-2 show an overall similar two-phase kinetics of expression suggests that this converging expression strategy might reflect important *in vivo* constraints for long term persistence in T-cells and evasion from the immune response which characterize these viruses. On the other hand we also observed some peculiar differences between HTLV-1 and HTLV-2 gene expression, especially concerning the relative expression and timing of the mRNAs coding for proteins that control Rex expression and function (see above). These differences might provide the molecular basis for the different tropism and pathogenicity of the two viruses.

However, in contrast to the other tumor viruses mentioned above, neither the levels or the timing of expression of different genes has been linked to a different disease outcome, a topic that should be addressed in future studies. In addition, both the “latent” expression detected in peripheral blood HTLV-infected cells and the “reactivation” upon culture *in vitro* are, at present, poorly understood phenomena. Further investigations are needed to define the pattern (and levels) of HTLV expression in different lymphoid organs.

Interestingly, also in the case of HIV-1, another complex human retrovirus, viral gene expression is controlled by two key regulatory proteins Tat and Rev, which control, respectively, transcription and post-transcriptional processing of alternatively-spliced viral mRNAs.

Early studies suggest that the HIV-1 life cycle is also characterized by a two-phase kinetics with Tat, Rev, and Nef expressed as early genes and Env, Vpu, Vif, Vpr, Gag, Pro, and Pol expressed as late genes. In the context of this expression strategy, Rev acts as a molecular switch controlling the transition between latent and productive infection (Ahmad et al., 1989; Kim et al., 1989). The two-phase kinetics was confirmed by Davis et al. (Davis et al., 1997), using cell-free HIV infection of Hut-78 cells. Using RT-PCR the authors observed the appearance of the tat, rev and nef mRNAs in the cytoplasm 12–16 h after infection, while env and gag expression reached its maximum between 20 and 24 h after infection. In contrast, cell-to-cell transmission of HIV-1 resulted in a late marked increase of env and gag mRNAs without a preceding peak of tat, rev and nef mRNAs.

However, to date, the relative abundance and the time course of expression has not been measured at a single transcript level using qRT-PCR like in the case of HTLV-1 and HTLV-2.

### Conflict of interest statement

The authors declare that the research was conducted in the absence of any commercial or financial relationships that could be construed as a potential conflict of interest.
